# Screening differential miRNAs responsible for permeability increase in HUVECs infected with influenza A virus

**DOI:** 10.1371/journal.pone.0186477

**Published:** 2017-10-23

**Authors:** Shujing Zhang, Ying Wu, Zinan Xuan, Xiaoming Chen, Junjie Zhang, Dongyu Ge, Xudan Wang

**Affiliations:** 1 Scientific Research Center, Beijing University of Chinese Medicine, Beijing, China; 2 Department of Microbiology and Immunology, School of Life Sciences, Beijing University of Chinese Medicine, Beijing, China; The University of Chicago, UNITED STATES

## Abstract

Severe influenza infections are featured by acute lung injury, a syndrome of increased pulmonary microvascular permeability. A growing number of evidences have shown that influenza A virus induces cytoskeletal rearrangement and permeability increase in endothelial cells. Although miRNA’s involvement in the regulation of influenza virus infection and endothelial cell (EC) function has been well documented, little is known about the miRNA profiles in influenza-infected endothelial cells. Using human umbilical vein endothelial cells (HUVECs) as cell models, the present study aims to explore the differential miRNAs in influenza virus-infected ECs and analyze their target genes involved in EC permeability regulation. As the results showed, permeability increased and F-actin cytoskeleton reorganized after HUVECs infected with influenza A virus (CA07 or PR8) at 30 MOI. MicroRNA microarray revealed a multitude of miRNAs differentially expressed in HUVECs after influenza virus infection. Through target gene prediction, we found that a series of miRNAs were involved in PKC, Rho/ROCK, HRas/Raf/MEK/ERK, and Ca^2^+/CaM pathways associated with permeability regulation, and most of these miRNAs were down-regulated after flu infection. It has been reported that PKC, Rho/ROCK, HRas/Raf/MEK/ERK, and Ca^2+^/CaM pathways are activated by flu infection and play important roles in permeability regulation. Therefore, the cumulative effects of these down-regulated miRNAs which synergistically enhanced activation of PKC, Rho/ROCK, Ras/Raf/MEK/ERK, and Ca^2+^/CaM pathways, can eventually lead to actin rearrangement and hyperpermeability in flu-infected HUVECs.

## Introduction

Influenza A virus is one of the most common respiratory pathogens responsible for considerable morbidity and mortality worldwide, particularly in children and the elderly population [1]. Severe influenza infections are featured by acute lung injury (ALI), a syndrome of increased pulmonary microvascular permeability that leads to hypoxia, respiratory failure, and even multiorgan failure (MOF) with vascular hyperpermeability [2–4]. Recently, a few studies have investigated the mechanisms through which influenza A virus may induce vascular hyperpermeability. It has been reported that apoptosis, degradation of the tight junction protein claudin-5 (Claudin-5), virus–cytokine-protease cycle, and phosphorylation of myosin light chain (MLC) may play certain roles in influenza–induced endothelial leak [5–7]. In our previous study, we have found that p38MAPK, Rho/ROCK and PKC pathways were involved in influenza-induced cytoskeletal changes and permeability increases in pulmonary microvascular endothelial cells (PMVECs) via phosphorylating ERM [8]. While it has been well documented that influenza infection can lead to endothelial hyperpermeability and the involved pathways have also been revealed, the elaborate cellular processes need to be further explored.

MicroRNAs (miRNAs) are small, endogenously produced non-coding RNAs, with 17–24 nucleotides in length. MiRNAs can regulate gene expression either by repressing mRNA translation or inducing mRNA degradation mostly through complementary binding to the 3’ untranslated regions (UTRs) of mRNAs [9]. A series of studies have demonstrated that host cellular miRNAs play important roles in the regulation of influenza A virus infection and replication in influenza-infected cells and patients [10–14]. MiRNAs also participate in the regulation of the vascular endothelial functions, such as TNF-alpha-, LPS-, or high-glucose-induced endothelia hyperpermeability [15–18]. Studies on miRNAs’ participation in virus-infected endothelia cells (ECs) abound. For instance, Pepini T, et al reported that Andes hantavirus (ANDV)’s infection of human endothelia cells resulted in changes in the expression of specific EC miRNAs, suggesting that ANDV infection interfered with key miRNA-regulated EC responses responsible for maintaining vascular integrity [19]. However, till now, the miRNAs which participate in influenza A virus induced-hyperpermeability have not been fully determined. In this study, therefore, we detected the differential miRNAs in human umbilical vein endothelial cells (HUVECs) after influenza A virus infection by using miRNA microarray and identified the miRNAs whose target genes were involved in the pathways regulating vascular permeability.

## Materials and methods

### Cells and virus

Human umbilical vein endothelial cells (HUVECs) were purchased from American Type Culture Collection (ATCC) and maintained in Endothelial Cell Medium (ECM, Sciencecell) supplemented with 1% Endothelial Cell Growth supplements (ECGS), 5% fetal calf serum serum (FCS, Sigma), 1% penicillin and 1% streptomycin. Madin Darby canine kidney (MDCK) cells were cultured in Dulbecco’s modified Eagle’s medium (DMEM, GIBCO) supplemented with 10% fetal bovine serum (FBS, Sigma), 1% penicillin and 1% streptomycin. The cells were cultured in a humidified atmosphere of 5% CO2 at 37°C.

Influenza A virus A/PR/8/34(H1N1) and A/Ca/07/2009(H1N1) were provided by Institute of Microbiology and Epidemiology, Academy of Military Medical Sciences, Beijing. Viruses were propagated in embryonated chicken eggs (1 to 3 passages), and samples of allantoic fluid were stored at -80°C. Virus titers were determined by plaque forming units in MDCKs using established protocols [20,21].

### Viral infection

When grown to 90% confluency, HUVECs were removed from the culture medium and washed twice with PBS. Cells were infected with influenza A virus in serum-free media at a multiplicity of infection (MOI) of 20~40 and harvested at the indicated time points. The non-infected cells were used as normal control.

### Immunofluorescence staining of influenza A virus

HUVEC monolayers in 24-well plates were uninfected or infected with PR8 or CA07 at a MOI of 20~40. At 12 or 24 hour after infection, the virus was removed from the HMVECs. The cells were washed with phosphate-buffered saline (PBS) for three times and then fixed in 30% ice-cold acetone for 1 hour at 4°C. Viral nucleoprotein (NP) protein was detected by using mouse monoclonal antibody (1:1000, 0.3 ml) for 60 minutes at 37°C(Chemicon International, Inc.). The secondary goat anti-mouse antibody labeled with fluorescence was used for 60 minutes at 37°C. After adding 4',6-Diamidino-2-Phenylindole, Dihydrochloride (DAPI, 1:10, 0.3 ml) and incubating for 10 minutes, the cells were washed with PBS for three times. Fluorescent images were acquired by MicroSuite FIVE software (Olympus Soft Imaging Solutions) with an Olympus BX61 motorized microscope (Olympus). The infection rate of HUVECs was calculated as the percentage of infected cells divided by the total cells.

### Transmonolayer electrical resistance (TER) of HMVEC monolayers

The permeability of HUVECs infected with PR8 or CA07 was detected by transmembrane resistance method at 0 h, 2 h, 4 h, 6 h, 12 h, 24 h and 36 h after infection. HUVECs were grown on 0.4um pore (Millipore) with 0.1ml media in the upper chamber and 0.6ml media in the lower chamber. Blank measurement was taken using a membrane insert with media only (no cells). Before the permeability of HUVECs was detected, the media were aspirated and the cells were washed with PBS twice. Then the ECM without serum was used to culture the cells.The HUVECs were subjected to the virus at a MOI of 30. Resistance measurements were corrected for the area of the membrane insert and blank measurements using the following formula: (R_experiment_-R_blank_)*area. A decrease in TER represented disruption of endothelial junctions and increased permeability, and increased TER suggested improved integrity of the cells.

### Confocal scanning laser microscopy

The distribution and morphology of F-actin were detected by confocal microscopy. HUVECs growing in 24-well plates were infected with PR8 or CA07 at a MOI of 30. At 12 h and 24 h after infection, the cells were rinsed quickly with PBS for 3 times and fixed with 4% paraformaldehyde at 4°C for 15 min. HUVECs were permeabilized at 4°C for 5 min with 0.25% Triton X-100 in 0.3 ml. After rinsing for 3 times with PBS, HUVECs were incubated with rhodamine phalloidin at 37°C for 40 min to label F-actin. Following incubation, cells were rinsed with PBS and incubated with DAPI for 10 min to stain nuclei. HUVECs were viewed with a confocal microscope (Olympus) using the filters for 384 and 470 nm.

### Flow cytometry detection of apoptosis

The apoptosis of HUVEC, at 24 h after CA07 or PR8 infection, was detected by TdT-mediated dUTP nick end labeling (TUNEL) method. Cells were prepared using the In Situ Cell Death Detection Kit, Fluorescein (Roche) according to the manufacturer’s instructions and analyzed by flow cytometry using a BD FACS Calibur cytometer (Becton Dickinson). Cells incubated with DNase I recombinant (3 U/ml in 50 mM Tris-HCl, pH 7.5, 1 mg/ml BSA) for 10 min at 15 ~ 25°C, were used as positive controls. Data analysis was performed with BD FACS Diva software and displayed as one-parameter histograms, with fluorescence intensity on the logarithmic *x*-axis versus percentage of total cell counts on the *y*-axis.

### RNA isolation and MicroRNA microarray analysis

Total RNA was extracted from uninfected and infected HUVECs using Trizol reagent (Invitrogen) following the manufacturer’s protocol. RNA pellets were resuspended in RNase-free water. RNA quantification was performed according to the Affymetrix recommended protocols. Total RNA was labeled using the FlashTag™ Biotin RNA Labeling Kit (Genisphere, Hatfield, PA). Briefly, the process begins with a brief poly(A) tailing reaction followed by ligation of the biotinylated signal molecule to the target RNA sample. The labeled miRNA was hybridized to Affymetrix® GeneChip® miRNA (Affymetrix miRNA 4.0) according to the manufacturer’s protocols. After washing and staining, the arrays were scanned with Affymetrix Scanner3000 (Affymetrix). Three numbers of replicates were conducted in each goup. The digitized image data was processed using Affymetrix GeneChip Command Console (version4.0, Affymetrix). Quality control analysis was performed using Expression Console (version1.3.1, Affymetrix)and analyzed using the Genespring software (version12.5, Agilent Technologies).

### Prediction of miRNA-binding sites and pathway analysis

Determination of potential miRNA targets was performed with Target Scan, PITA, and microRNAorg (28, 29). Pathway analysis to identify functions of miRNA target genes was performed using GO and KEGG enrichment.

### Reverse transcription, real-time PCR, and miRNA validation

Total RNA (2μg) was reverse transcribed using the miRcute miRNA First-Strand cDNA Synthesis Kit (TIANGEN)using a miRNA-specific primer (37°C for 60 min). MiRNA-specific cDNAs were used as templates for real-time PCR using the miRcute miRNA qPCR Detection Kit (SYBR Green, TIANGEN) according to the manufacturer’s protocol. cDNA and SYBR green PCR master mix were diluted and aliquoted into each well of a PCR array in the Applied Biosystems 700 RT-PCR machine. Real-time PCR was performed using the following thermos-cycling parameters: 1 cycle of 95°C for 5 min and then 95°C for 15 s, 60°C for 20 s, and 72°C for 30 s for a total of 40 cycles. The threshold cycle of each miRNA from virus-infected and uninfected samples was normalized to that of U6 small nuclear RNA levels. The fold change in expression levels of each miRNA was determined by comparing the expression levels of miRNAs within virus-infected and uninfected HUVECs. F equation, as expressed by 2^-ΔCt^, is used to calculate the relative amount of the miRNAs, as compared with the housekeeping gene in each group. F’ equation, as expressed by 2^-ΔΔCt^, is used to calculate the relative amount of the miRNAs in the infected group, as compared with that in the non-infected group. Microarray data were validated by analysis of individual miRNAs using RT-PCR in triplicate. Primers for stem-loop or adding-tail PCR were listed as follows: has-mir-138-5p-F, 5’- CTGGTGTTGTGAATCAGGCCG-3’; hsa-miR-99a-5p, RT primer, 5’- GTCGTATCCAGTGCAGGGTCCGAGGTATTCGCACTGGATACGACcacaaga-3’, forward, 5’- GCCCGTCCGATCTTGTGAA-3’; reverse, 5’- GTGCAGGGTCCGAGGT-3’; hsa-miR-147b-F, 5’- TGTGCGGAAATGCTTCTGCTA’; hsa-miR-181a-5p-F, 5’- AACATAGAACGCTGTCGGTGA-3’; hsa-miR-21-5p-F, 5’- CGCTAGCTTATGAGACTGATGTTGA-3’; hsa-miR-200a-3p-F, 5’- GGCTAACACTGTCAGGTAACGATGT-3’; hsa-miR-29b-1-5p-F, 5’-GCTGGTTTCATATGGTGGTTTAGA-3’; hsa-miR-29a-3p-F, 5’- CCCGTAGCACCATCTGAAATAA-3’; hsa-miR-23a-5p-F, 5’- GGTTCCTGGGGATGGGATTT-3’; hsa-miR-501a-3p-F, 5’- AATGCACCCGGGCAAGGATTCT-3’; hsa-miR-29b-5p-F, 5’- GCTGGTTTCATATGGTGGTTTAGA -3’; hsa-miR-21-3p-F, 5’-CAACACCAGTCGATGGGCTGT-3’; hsa-miR-U6-F, 5’- CAAGGATGACACGCAAATTCG-3’; has-miR-4485-F, 5’-TAACGGCCGCGGTACCCTAA-3’.

### Statistical analysis

Significance of differences among two groups was tested by a Student’s t test. And among more than two groups, one-way analysis of variance (ANOVA) was employed. P values<0.05 were considered to be statistically significant. All results were computed using Prism version 5.0c and are presented as mean ± SEM.

## Results

### Infection rate of influenza A virus on HUVECs

**Fig 1:**
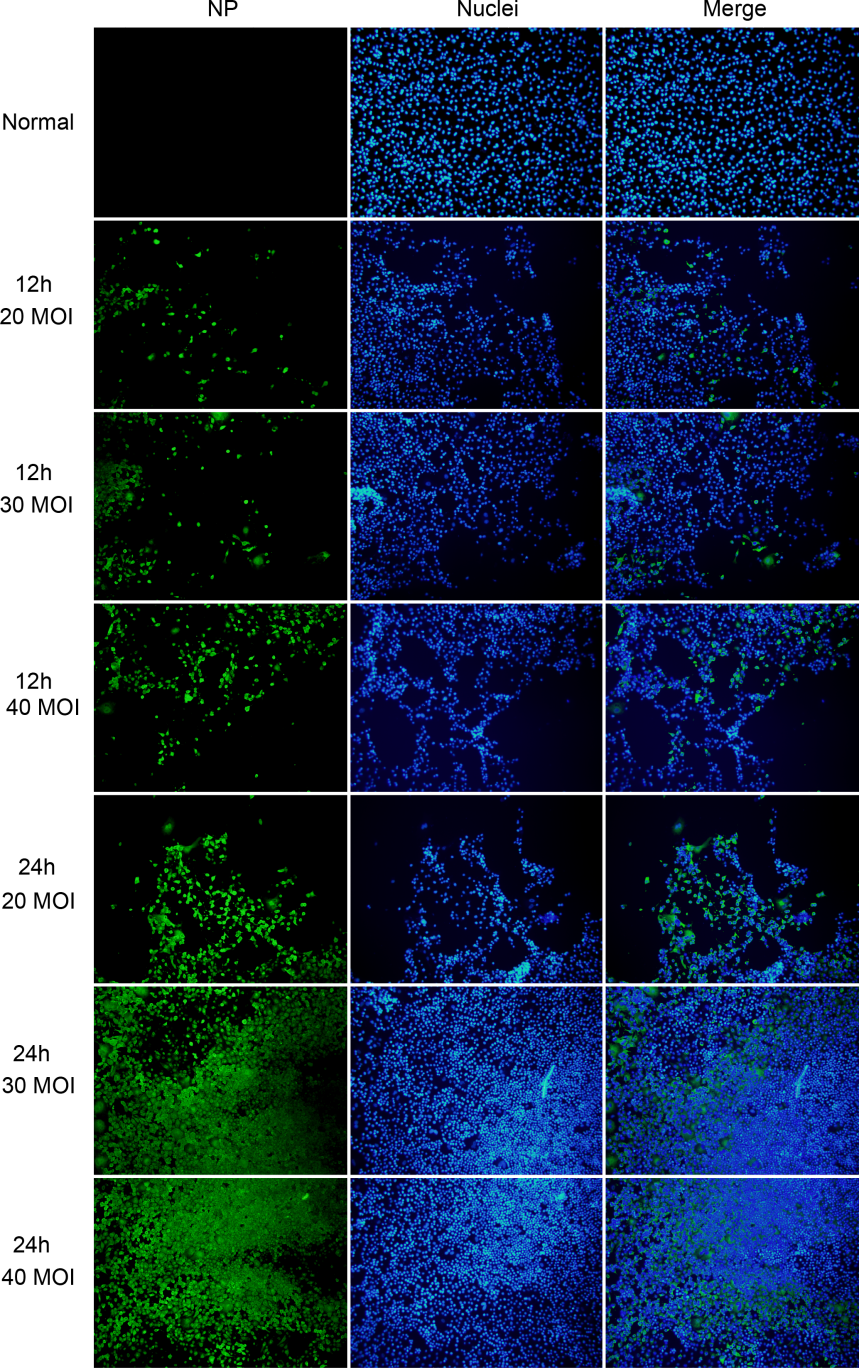
The infection rate of HUVECs was determined by immunofluorescence staining of nucleoprotein (NP) protein. The immunofluorescence staining of normal cell, The infection rate of PR8 at MOI of 20, 30 and 40 were 32%, 46%, 51%, respectively at 12 h after infection and 50%, 62%, 68%, respectively at 24 h after infection. NP (green), nuclei (blue) were examined using fluorescent microscope.

The infection rate of influenza A virus in HUVECs was detected to ensure miRNA expression alteration and permeability change of the cells was caused by the intracellular virus. The immunofluorescence staining of normal cell and indirect immunofluorescence showed the infection rate of PR8 at MOI at 20, 30 and 40 were 32%, 46%, 51%, respectively at 12 h after infectionwhile the infection rate was 50%, 62%, 68%, respectively at 24 h after infection (Fig 1). Although PR8 at a MOI of 40 showed a higher infection rate, the HUVECs had lost their normal morphology during infection. Thus, virus at a MOI of 30 was adopted in the following experiments. Likewise, CA07 shared a similar infection rate compared with PR8.

### Influenza A virus infection induced hyperpermeability and F-actin changes in HUVECs

**Fig 2:**
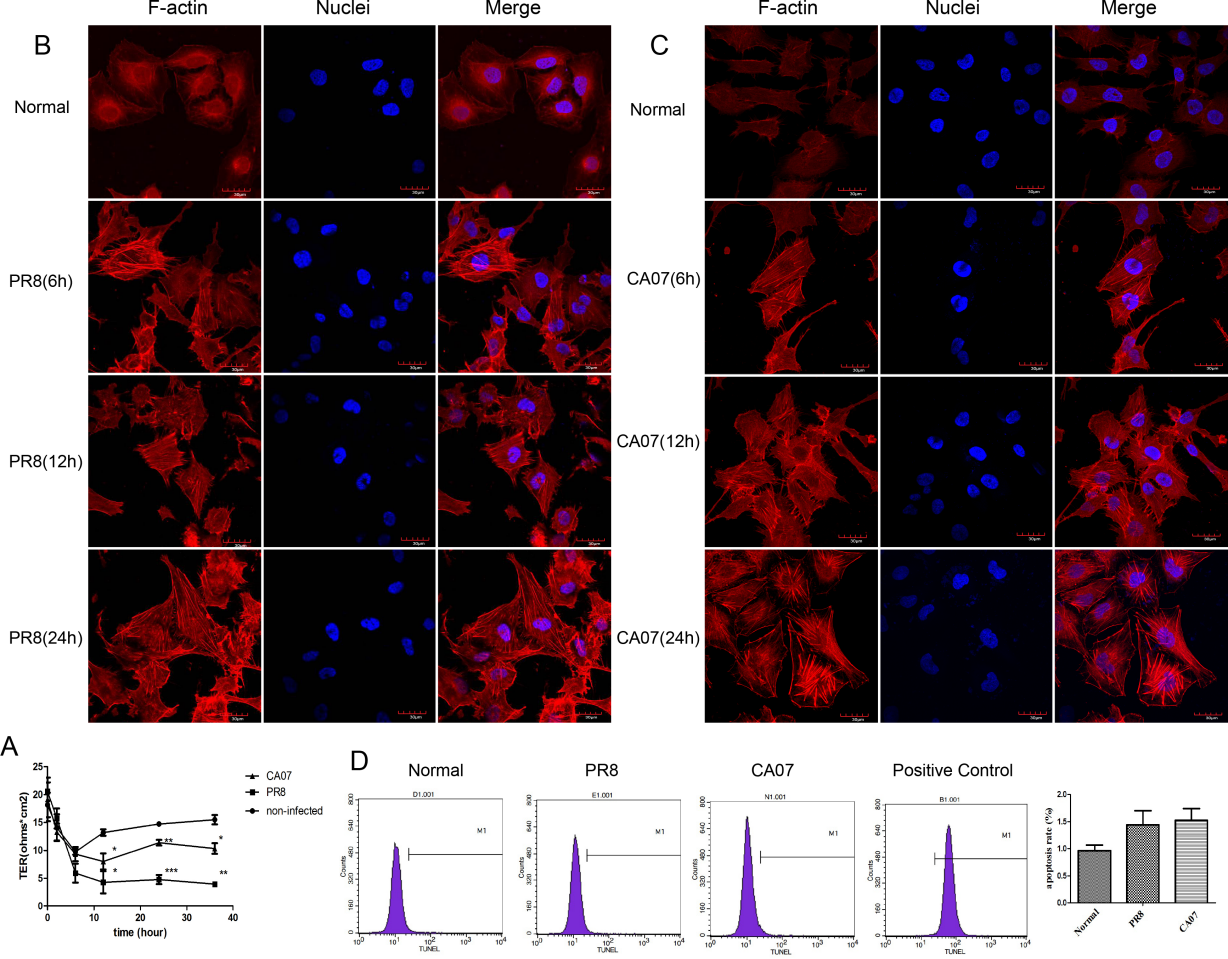
Effect of influenza A virus on transmonolayer electrical resistance (TER) and cytoskeleton in HUVECs. (A) HUVEC monolayers were infected with PR8 or CA07 at a MOI of 30, and the non-infected cells were used as control. TER values were monitored at 0 h, 2 h, 4 h, 6 h, 12 h, 24 h and 36 h after infection. *P< 0.05 versus non-infected cells, **P< 0.01 versus non-infected cells, ***P< 0.001 versus non-infected cells. (B) At 6, 12, 24 h after 30 MOI of PR8 or (C) CA07 infection, F-actin in HUVECs were examined using confocal microscopy with non-infected cells as control. F-actin cytoskeleton (red) and nuclei (blue) were examined using confocal microscopy. Scale bars represent 30um. PR8 and CA07 infection caused F-actin rearrangement in HUVECs. (D) Apoptosis of HUVECs were determined by TUNEL method via flow cytometry. The average fluorescence intensity was calculated by the Image software from three independent experiments. Three replicates were included. Data are expressed as means ± SEM, n = 3. The flow cytometry showed no significant apoptosis in HUVECs infected by PR8 or CA07.

TER began to decrease at 6 h after PR8 or CA07 challenge, which showed permeability increases. At 24 h post infection, TER in infected cells decreased significantly compared with that in uninfected cells (Fig 2A). There were the TER decreases both in the non-infected and infected cells at the early stage post infection. We speculated the procedures of washing cells and replacing media before TER detection might be responsible for the permeability increases, which would be a stimulation to HUVECs. Meanwhile, we also observed cytoskeletal reorganization in HUVECs after virus infection, which is accompanied by TER decrease. At 6 h, 12 h, 24 h post infection, confocal microscopy demonstrated F-actin thickening and bundling in the cytoplasm of HUVECs with the time prolongation, and PR8 infection showed more obvious changes in TER decreases and stress fiber formation, compared with that of CA07 (Fig 2B and 2C). In order to explore the possible association between apoptosis and virus-induced hyperpermeability, the apoptosis of HUVECs was detected by flow cytometry. The apoptosis rates were 0.9633 ± 0.09838 in uninfected cells, and 1.440 ± 0.2593, 1.530 ± 0.2088, respectively, in PR8 and CA07 infected cells. The data showed no significant difference between the uninfected cells and PR8 or CA07 infected cells (Fig 2D).

### MicroRNA expression changed in HUVECs during influenza A virus infection

**Fig 3:**
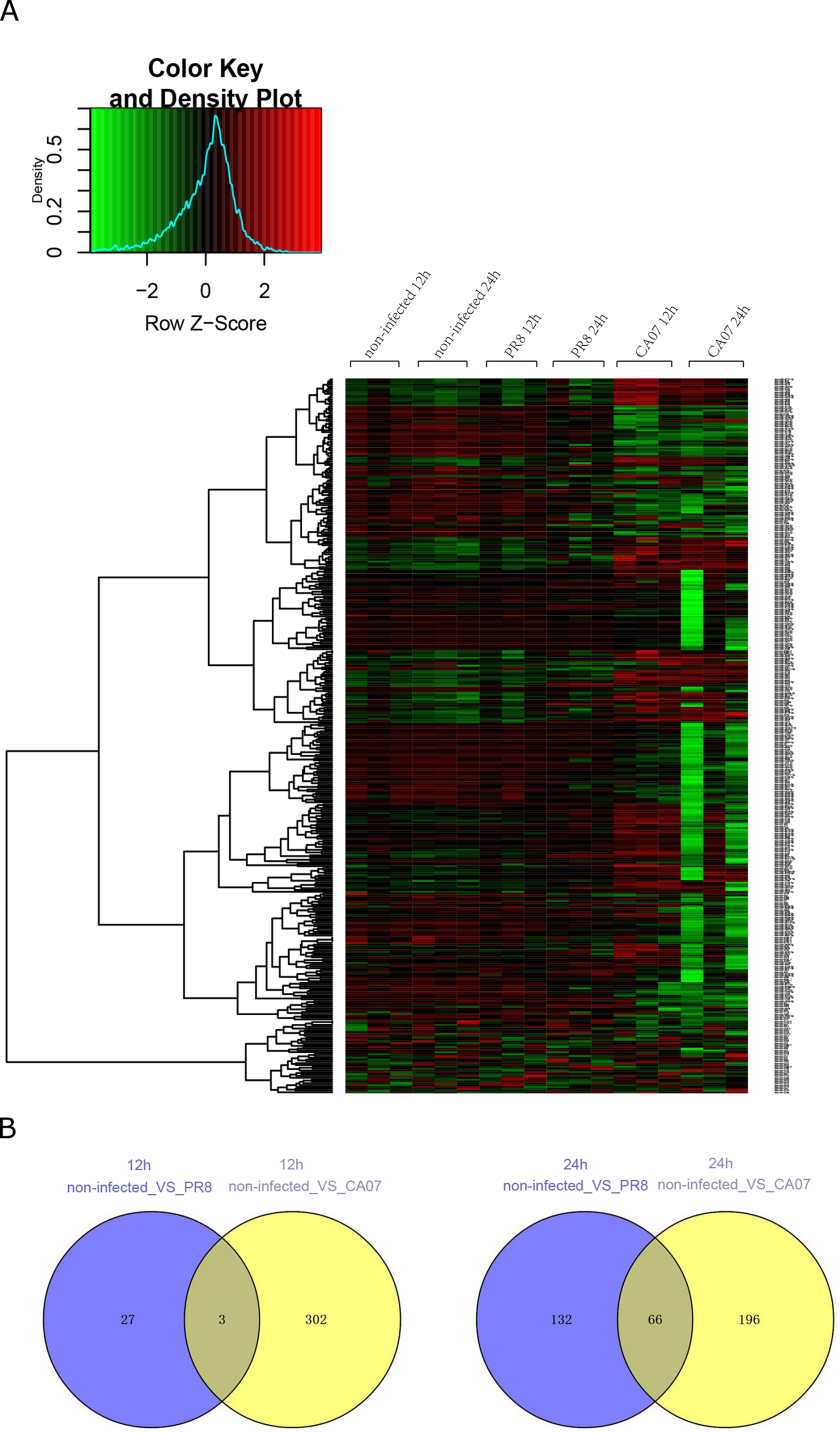
Analysis of miRNA profiles in HUVECs infected with influenza A virus. (A) At 12 h and 24 h after PR8 infection or CA07 infection, miRNA microarray was used to analyze the differential miRNAs between the miRNA profiles of the flu-infected and the non-infected cells. The up-regulated genes were shown in red and the down-regulated were shown in green. Three numbers of biological replicates were conducted. A1 and A2 indicated the miRNAs in non-infected cells at 12 h and 24 h, respectively; B1 and B2 indicated the miRNAs in PR8-infected HUVECs at 12 h and 24 h after infection, respectively; C1 and C2 indicated the miRNAs in CA07-infected HUVECs at 12 h and 24 h after infection, respectively. The degree of similarity between the replicates were demonstrated by Pearson's Correlation Coefficient, with the r values ranged from 0.77 to 0.99. (B) The numbers of coinciding miRNA that differentially expressed at the same time point in PR8- or CA07- infected- HUVECs is shown at the junction of two circles.

To determine whether influenza A virus infection could alter cellular miRNAs expression in HUVECs, two common laboratory strains of influenza A virus, PR8 and CA07 were applied. A microarray representing a total of 2,578 human mature miRNAs was used to detect microRNA expression. By comparing the abundance of cellular miRNAs in influenza A virus-infected cells with those in uninfected cells, microRNAs that exhibited differences in abundance with a comparative p value of < 0.05 were determined as differential miRNAs. The data showed the abundance of 30 miRNAs dramatically altered at 12 h after PR8 infection, among which 17 miRNAs down-regulated and 13 miRNAs up-regulated. The number of differentially expressed miRNAs increased with the time. At 24 h post PR8 infection, the levels of 198 miRNAs changed dramatically versus uninfected ECs: 126 miRNAs decreased and 72 increased (Fig 3A). There were 4 miRNAs, hsa-miR-3162-5p, hsa-miR-5189-5p, hsa-mir-6723, and U71b, displaying difference in both time points after influenza A virus infected.

Interestingly, for CA07-infected cells, more miRNAs exhibited significant changes in expression level, compared with PR8-infected samples. There are 305 miRNAs expressed differentially at 12 h post infection, among which 146 miRNAs down-regulated and 159 up-regulated. At 24 h after infection, 262 miRNAs showed dramatic difference in abundance, with 198 decreased and 64 increased. 129 miRNAs showed consistent changes in abundance at 12 and 24 h post infection, such as hsa-let-7i-5p, hsa-miR-23a-3p, hsa-miR-31-5p, has-miR-16-5p, has-miR-181a-5p, has-miR-149-3p, has-miR-20a-5p, has-miR-221-3p, has-miR-222-3p, has-miR-24-3p, has-miR-103a-3p, has-miR-361, etc (Fig 3A).

It was found that there were 3 miRNAs, hsa-miR-5196-5p, hsa-miR-6716-5p, U34, and 66 miRNAs, exemplified by hsa-miR-23a-3p, hsa-miR-181a-5p, etc, showing the same fluctuations between PR8- and CA07- infected HUVECs at 12 h post-infection and 24 h post-infection, respectively (Fig 3B).

### Target prediction of miRNA expression profile

The Targetscan, PITA, and microRNAorg softwares were used to predict the miRNA-target genes, and then KO and KEGG enrichment were used to analyze the regulated pathways. The results showed that miRNAs corresponded to multiple target genes and were involved in the regulation of varied pathways, including inflammatory response, cytoskeletal formation, anti-viral response and apoptosis pathway, etc. Recent studies reported that besides apoptosis, Ca^2+^-CaM, Rho/ROCK, Ras/Raf/MEK/ERK and PKC pathways were all involved in the regulation of microfilament assembly or contractility, thereby resulting in increased permeability. Influenza A virus infection can induce phosphorylation MLC by activating PKC, Ca^2+^-CaM, Ras/Raf/MEK/ERK and ROCK pathways, which lead to F-actin reorganization and permeability increases. Our previous study showed that Rho/ROCK and PKC were involved in influenza-induced cytoskeletal changes and permeability increases in HUVEC via phosphorylating ERM. Accordingly, our study primarily focused on the miRNA-target genes involved in PKC, Rho/ROCK, MLC, Ras/Raf/MEK/ERK, F-actin, Ca^2+^ pathways regulating cytoskeletal rearrangement and permeability regulation. The differential miRNAs and their target genes in the above pathways were concluded in Tables 1 and 2, and the number of up-regulated or down-regulated miRNAs targeting at these pathways were displayed in Table 3, which suggested that the down-regulated miRNAs outnumbered the up-regulated miRNAs.

**Table 1:** List of the differential miRNAs and their target genes in HUVECs at 12 h and 24 h after PR8 infection.

PR8 12-hour				PR8 24-hour				PR8 24-hour			
Mi RNA	Fold Change	FDR p-value	Target Gene	Mi RNA	Fold Change	FDR p-value	Target Gene	Mi RNA	Fold Change	FDR p-value	Target Gene
miR-92a-1-5p	1.21	1.16E-02	PRKCB,CAMK2A,GSK3B,HRAS	miR-663a	1.88	5.61E-03	ROCK2,PRKCB	miR-26a-5p	-1.34	3.01E-02	GSK3B
miR-501-3p	-1.12	1.27E-02	CAMK2B,NRAS,HRAS	miR-149-3p	1.31	2.89E-02	PRKCG,HRAS	miR-27a-3p	-1.79	9.79E-03	ACTA2
				let-7b-5p	-1.13	4.39E-02	CAMK2B	miR-27b-3p	-1.43	2.78E-02	ACTA2
				miR-126-3p	-1.64	4.95E-02	PRKC	miR-29b-1-5p	-3	1.63E-03	CAMK2D
				miR-130a-3p	-1.34	4.62E-02	PRKCB,HRAS	miR-30a-5p	-1.59	4.01E-02	MYL6
				miR-138-5p	-1.41	3.44E-04	CAMK2D	miR-30c-5p	-1.46	2.02E-02	MYL6
				miR-147b	-3.29	1.71E-02	ROCK2	miR-31-5p	-1.12	1.89E-02	MYL6
				miR-151a-3p	-1.37	3.95E-02	MYL6,MYL9	miR-34a-5p	-1.55	4.81E-02	MYL9
				miR-151a-5p	-1.14	1.22E-02	MYL6	miR-423-3p	-1.28	2.50E-02	MYL6,MYL9,CLDN5,HRAS
				miR-181a-5p	-1.41	2.31E-02	CAMK2D,CLDN5	miR-424-3p	-1.97	5.30E-05	ROCK1,GSK3B
				miR-181b-5p	-1.42	9.30E-05	ACTA2,MYL6	miR-502-3p	-1.34	5.51E-03	NRAS
				miR-188-5p	-2.94	4.45E-02	ACTA2	miR-503-5p	-1.38	1.91E-02	PRKCG
				miR-18a-5p	-1.5	1.88E-02	CAMK2A	miR-532-5p	-1.32	7.27E-03	CAMK2D
				miR-193b-5p	-1.23	4.64E-02	CAMK2D	miR-615-3p	-1.58	4.85E-02	PRKCG
				miR-200a-5p	-2.4	1.18E-02	HRAS	miR-652-3p	-1.28	3.00E-02	ROCK1
				miR-21-3p	-2.1	1.89E-03	HRAS	miR-744-5p	-1.13	4.09E-02	MYL6,CLDN5
				miR-23a-3p	-1.12	1.55E-02	MYL6	miR-769-3p	-1.42	3.32E-02	CAMK2G
				miR-23a-5p	-5.17	1.11E-03	ROCK1	miR-941	-1.74	1.17E-02	CLDN5
				miR-24-3p	-1.16	2.82E-02	HRAS				

**Table 2:** List of the differential miRNAs and their target genes in HUVECs at 12 h and 24 h after CA07 infection.

CA07 12-hour				CA07 12-hour				CA07 24-hour			
Mi RNA	Fold Change	FDR p-value	Target Gene	Mi RNA	Fold Change	FDR p-value	Target Gene	Mi RNA	Fold Change	FDR p-value	Target Gene
miR-100-5p	1.75	1.10E-05	CAMK2D	miR-193b-5p	-2.74	5.56E-04	HRAS	miR-663a	6.36	1.90E-05	PRKCG,HRAS
miR-663a	3.71	2.01E-03	PRKCG,HRAS	miR-200a-3p	-3.84	1.30E-03	CAMK2B	miR-149-3p	2.4	1.92E-03	CAMK2B,HRAS
miR-149-3p	1.97	2.78E-03	CAMK2B,HRAS	miR-200a-5p	-2.47	8.47E-04	HRAS	miR-638	1.53	1.23E-02	CAMK2B,HRAS
miR-885-3p	1.78	7.83E-03	PRKCG	miR-210-3p	-3.95	7.67E-04	CAMK2G	let-7i-5p	-1.86	6.97E-03	PRKCB1
miR-494-3p	2.29	1.31E-02	CAMK2D	miR-212-3p	-1.79	4.46E-02	MYL9,MYC	miR-125a-5p	-1.49	2.80E-02	MYL9
miR-484	1.78	2.48E-02	PRKCB1	miR-21-3p	-2.34	2.38E-02	MYL6	miR-126-3p	-22.7	2.00E-02	PRKCB,HRAS
miR-638	1.85	3.84E-02	CAMK2B,CASP9,HRAS	miR-23a-3p	-1.92	4.20E-03	ROCK1	miR-132-3p	-1.91	4.21E-03	NRAS
let-7i-5p	-1.8	4.11E-02	PRKCB1	miR-24-3p	-1.36	1.09E-02	HRAS	miR-138-5p	-12.08	1.85E-02	ROCK2
miR-125a-5p	-1.76	1.19E-02	MYL9	miR-26a-5p	-2.22	2.55E-03	GSK3B	miR-146a-5p	-1.99	1.90E-04	ACTA2,ROCK1
miR-126-3p	-2.21	1.11E-02	PRKCB,HRAS	miR-27a-3p	-2.63	1.48E-03	ACTA2	miR-147b	-6.25	4.54E-02	MYL6,MYL9
miR-130a-3p	-2.98	1.28E-03	CAMK2D	miR-27b-3p	-2.97	1.49E-03	ACTA2	miR-149-5p	-13.27	3.96E-02	HRAS
miR-132-3p	-1.88	8.05E-03	NRAS	miR-28-5p	-1.5	2.10E-02	CAMK2D	miR-181a-5p	-1.95	1.29E-02	ACTA2,MYL6
miR-138-5p	-2.18	1.71E-03	ROCK2	miR-29a-3p	-1.89	1.67E-02	CAMK2B	miR-181b-5p	-1.84	3.26E-04	ACTA2
miR-149-5p	-2.3	1.52E-02	HRAS	miR-29b-3p	-4.39	1.09E-02	CAMK2B	miR-191-5p	-1.34	6.76E-03	PRKCB1
miR-15b-5p	-2.8	4.98E-02	PRKCD	miR-29c-3p	-6.29	3.03E-02	CAMK2B	miR-21-5p	-2.31	5.64E-03	ACTA2,ROCK1
miR-181a-5p	-2.1	2.54E-02	ACTA2,MYL6	miR-30b-5p	-9.08	8.08E-03	CAMK2B	miR-23a-3p	-1.95	2.13E-03	ROCK1
miR-181b-5p	-1.75	1.88E-02	ACTA2	miR-30c-5p	-3.9	2.95E-03	MYL6	miR-23b-3p	-1.86	1.73E-02	ROCK1
miR-181c-5p	-4.48	1.08E-02	ACTA2,GSK3B,KRAS	miR-31-5p	-1.98	5.38E-03	MYL6	miR-24-3p	-1.41	2.07E-03	HRAS
miR-181d-5p	-3.82	1.05E-02	ACTA2,GSK3B	miR-34a-5p	-6.18	8.48E-03	MYL9	miR-26a-5p	-2.42	3.36E-04	GSK3B
miR-182-5p	-1.56	2.91E-02	PRKCB1	miR-361-5p	-1.45	7.03E-03	PRKCB1	miR-27a-3p	-2.37	1.05E-02	ACTA2
miR-188-5p	-2.98	1.95E-02	CAMK2A	miR-424-3p	-3.21	1.65E-02	ROCK1,GSK3B	miR-29a-3p	-2.12	1.82E-03	CAMK2B
miR-191-5p	-1.33	4.23E-02	PRKCB1	miR-574-3p	-2.09	3.68E-02	PRKCB1,GSK3B	miR-30a-5p	-2.66	2.37E-02	MYL6
								miR-31-5p	-1.85	3.00E-05	MYL6
								miR-361-5p	-1.55	1.80E-03	PRKCB1
								miR-362-5p	-7.66	4.92E-02	ACTG2
								miR-99a-5p	-7.2	3.18E-02	CAMK2D
								miR-99b-5p	-1.57	1.95E-02	CAMK2D

**Table 3:** The number of up- and down-regulated miRNAs which target at Ca2+-CaM, Rho/ROCK, Ras/Raf/MEK/ERK and PKC pathways.

time	virus	MLC		CaMKII		actin		PKC		ROCK		CLAUDIN-5		RAS	
		up	down	up	down	up	down	up	down	up	down	up	down	up	down
12 h	PR8	0	0	1	1	0	0	1	0	0	0	0	0	1	1
	CA07	0	6	4	9	0	6	3	7	0	3	0	0	3	7
24 h	PR8	0	9	1	7	0	4	2	4	1	4	0	4	1	7
	CA07	0	5	2	3	0	6	2	4	1	5	0	0	3	4

### Confirmation of miRNA expression profile by qRT-PCR

**Fig 4:**
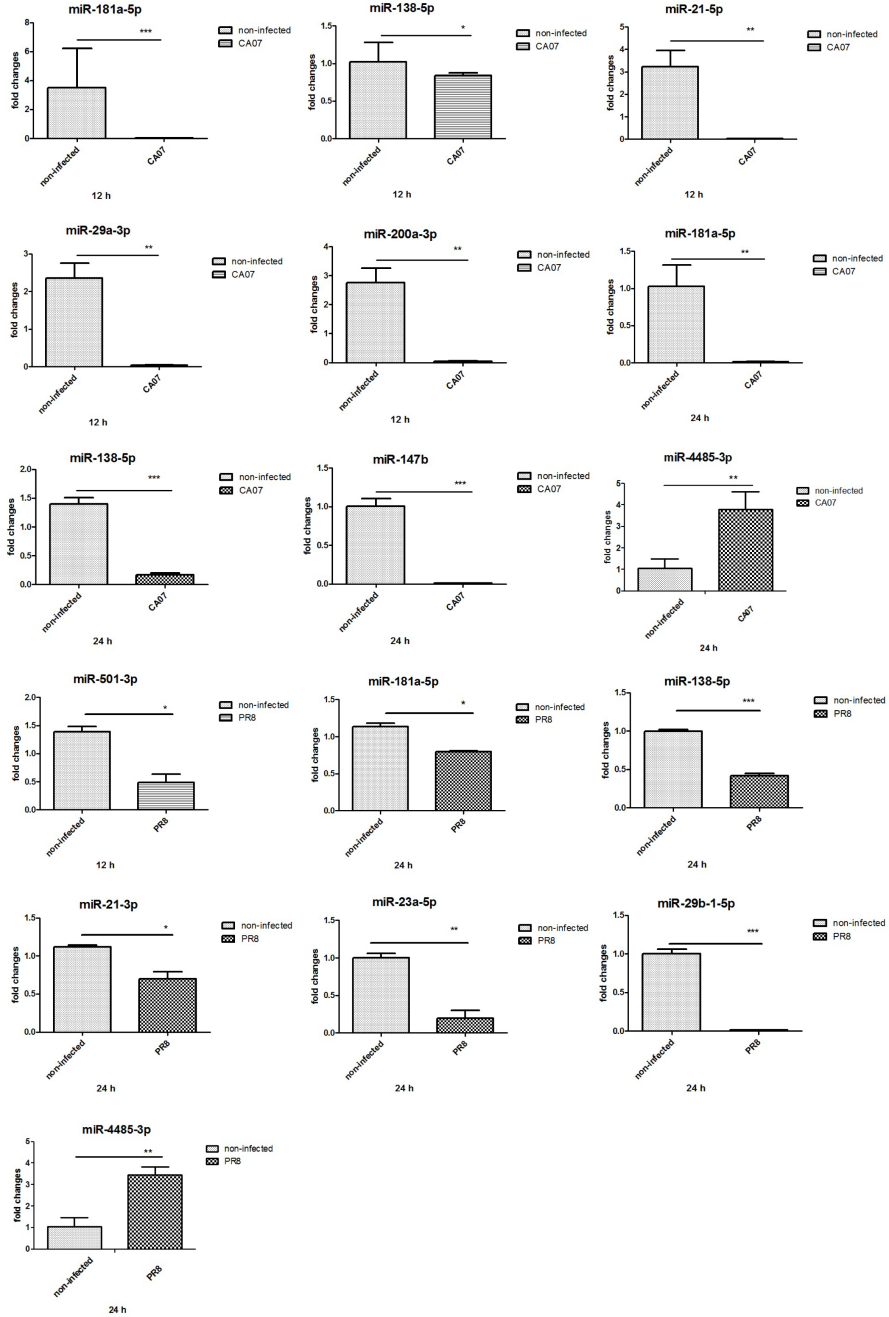
qRT-PCR measured the relative abundance of some miRNAs at 12 and 24 h after CA07 or PR8 infection. The relative abundance of each miRNA was determined in triplicate at different time points. *P< 0.05 versus non-infected cells, **P< 0.01 versus non-infected cells, ***P< 0.001 versus non-infected cells.

According to the results of target gene prediction, we chose some miRNAs whose target genes were participated in the regulation of hyperpermeability, to verify their changes in abundance by quantitative RT- PCR. There were general consistency between qRT-PCR assays and microarray results. At 12 h post infection, has-miR-181a-5p (*P < 0*.*001*), has-miR-138-5p (*P< 0*.*05*), has-miR-21-5p (*P < 0*.*01*), has-miR-29a-3p (*P < 0*.*01*), and has-miR-200a-3p (*P < 0*.*01*) were found to be decreased significantly in CA07-infected cells compared with those in non-infected cells; at 24 h, has-miR-181a-5p (*P < 0*.*01*) and has-miR-138-5p (*P < 0*.*001*), has-miR-147b (*P < 0*.*001*) were down-regulated in CA07-infected cells. For PR8-infected cells, at 12 h post infection, has-miR-501-3p (*P< 0*.*05*) was decreased significantly compared with that in non-infected cells; has-miR-181a-5p (*P < 0*.*05*), has-miR-138-5p (*P< 0*.*001*), has-miR-21-3p (*P < 0*.*05*), has-miR-23a-5p (*P < 0*.*01*), and has-miR-29b-1-5p (*P< 0*.*001*), were found to be decreased significantly, compared with that in non-infected cells at 24 h post infection. Has-miR-4485 in CA07-infected cells (*P < 0*.*01*) and PR8- infected cells (*P < 0*.*01*) were up-regulated at 24 h post infection (Fig 4).

## Discussion

Recent studies have suggested that influenza A virus induces cytoskeletal rearrangement and permeability increase in endothelial cells [22]. Although miRNA’s involvement in EC’s permeability regulation has been well documented, little is known about the miRNA profiles in influenza-infected ECs. Therefore, our study aims at revealing changes of miRNA abundance in ECs infected with influenza virus and selecting those whose target genes are involved in permeability regulation pathways.

HUVECs are commonly used cell models to study EC’s functions. However, unlike MDCKs or human respiratory epithelial cells which are susceptible to influenza infection, HUVECs show a relatively lower susceptibility according to Haidari et al [7]. To ensure the changes of miRNA responsible for HUVEC function are triggered by the intracellular virus, we detected the infection rate of HUVECs at 12 h and 24 h after the challenge of influenza A virus. As our results showed, the cells infected with PR8 at a MOI of 30 showed a higher infectivity rate. However, morphological changes marked by cell rounding and cell detachment, indicating cell death, have been observed in PR8-infected-cells at a MOI of 40. Using TER method and confocal microscopy, we demonstrated that 30 MOI of influenza A virus significantly evoked an increase in EC permeability, accompanied by rearrangement of F-actin cytoskeleton with thickening of F-actin bundling in the cytoplasm of HUVECs. Consistent with previous reports, our study also suggested that influenza virus could induce endothelial barrier dysfunction in ECs without significant cytotoxic effects, which was demonstrated by flow cytometry excluding apoptosis of HUVECs [5]. We noticed that remodeling of the actin cytoskeleton was observed in 10 MOI of influenza virus A/HK/2/68 (H3N2) infected-HUVECs and 1 MOI of Avian influenza virus subtype H9N2 (A/Quail/HK/G1/97) induced HUVECs apoptosis was reported by Chan et al [7,23]. However, when we infected HUVECs with influenza virus at 1 or 10 MOI, PR8 and CA07, showed a low infection rate without significant effect on ECs’ permeability and fiber formation (data no shown). We therefore speculated that the inconsistence was caused by different flu strains used in experiments. The influenza virus strains with severe pathogenesis in clinic could lead to more significant endothelial dysfunction.

Using MicroRNA Microarray, we examined the global patterns of cellular miRNA expression in HUVECs after influenza A virus infection. The major observations from this work were that influenza A virus infection resulted in the altered expression of cellular miRNAs in HUVECs, which relied heavily on the time duration. More miRNAs showed alterations in abundance as time accumulated and some miRNAs changed consistently under stimulation of the two virus strains or at both timepoints. In order to ascertain the miRNAs participating in permeability regulation of HUVECs, we took advantages of Targetscan, PITA, microRNAorg softwares and KO/ KEGG enrichment analysis to predict the miRNA target genes and found a mulitude of miRNAs were involved in permeability regulation pathways.

It is well known that contraction of the actin cytoskeleton which is intimately associated with permeability increase, are primarily regulated by phosphorylation of the regulatory subunit of MLC [24,25]. And it is well documented that influenza infection induces activation of the monomeric GTP binding protein RhoA, protein kinase C (PKC), and Ras/ Raf/MEK/ERK signaling cascade [26,27]. Activation of RhoA leads to the stimulation of Rho-kinase (ROCK), which, in turn, phosphorylates the regulatory myosin-binding subunit of MLC phosphatase (MLCP), resulting in the inhibition of MLCP, and then the phosphorylation of MLC. Activation of PKC and Ras/Raf/MEK/ERK signaling cascade inhibits MLCP and enhances MLC phosphorylation [28]. Meanwhile, influenza infection also leads to an influx in intracellular calcium (Ca^2+^) and actin polymerization [29–33]. These studies suggest that Ras/Raf/MEK/ERK, PKC, RhoA/ROCK pathways are activated after influenza infection and involved in influenza-induced actin cytoskeleton remodeling and permeability increase by phosphorylation of MLC. Our previous study also provided evidence for the involvement of Rho/ROCK and PKC pathways in influenza-induced hyperpermeability in microvascular cells via phosphorylating Ezrin/Radixin/Moesin (ERM). In a summary, these studies suggest that influenza virus infection activates PKC, Rho/ROCK, Ras/Raf/MEK/ERK, and Ca^2+^/CaM pathways contributing to endothelial dysfunction.

In the list of miRNA profiling in HUVECs infected by influenza virus, we found a series of miRNAs were involved in permeability regulation through their target genes in PKC, Rho/ROCK, HRas/Raf/MEK/ERK, and Ca^2+^/CaM pathways. For example, miR-181a-5p, targeting actin and MLC, was down-regulated at 12 h in CA07- infected HUVECs and at 24 h in CA07- and PR8- infected HUVECs; miR-147b, targeting MLC, was dramatically decreased by 6.25~ fold at 24 h after CA07 infection, which has been reported to protect endothelial barrier from LPS-induced inflammation; miR-21-5p, was found to be down-regulated at 12 h post CA07 infection, targeted ROCK1; miR-200a-3p, whose target gene was CAMK2B, was decreased at 12 h post CA07 infection and miR-29a-3p, targeting at CAMK2B and MYL6, was down-regulated by 1.89 fold at 12 h after CA07 infection; miR-501-3p was decreased about 1.2 fold at 12 h after PR8 infection, with Ras, and CaMKII as target genes; miR-29b-5p, whose target genes were CAMKII, was down-regulated by approximately 3 fold in PR8 infected HUVECs; miR-21-3p was decreased at 24 h after PR8 infection, with MLC as target gene; miR-23a-5p was decreased at 24 h after PR8 infection, with HRAS as target gene. qRT-PCR demonstrated these miRNAs’ alterations were generally consistent with microarray data.

The miRNA profiling list showed most miRNAs whose target genes were key molecules in PKC, Rho/ROCK, Ras/Raf/MEK/ERK, and Ca^2+^/CaM pathways, were down-regulated after influenza virus infection, causing activation of these pathways, then leading to actin cytoskeleton remodeling and permeability increase. We did not find a specific miRNA which could regulate EC permeability at both timepoints after the two strains of influenza virus infection, therefore, we speculated that actin rearrangement and hyperpermeability in flu-infected HUVECs were the cumulative effects of these down-regulated miRNAs which synergistically enhanced activation of PKC, Rho/ROCK, Ras/Raf/MEK/ERK, and Ca^2+^/CaM pathways.

Taken together, the present study provided a global miRNA profiling in influenza virus infected-HUVECs and indicated a series of miRNAs contributing to influenza virus-induced EC hyperpermeability via regulating their target genes in the upstream pathways of permeability regulation. Our study suggests that influenza virus, like hantavirus, can induce EC permeability increase by adjusting miRNA abundance, thereby causing the target genes and pathways activation. Based on these results, further studies are warranted to explore and validate the functions of the differential miRNAs on EC permeability and actin remodeling.
